# Gastric adenocarcinoma in Situs inversus totalis: a case study and literature review

**DOI:** 10.3389/fonc.2023.1238467

**Published:** 2023-10-25

**Authors:** Bo Sun, Ping Xu, Pengfei Kong, Yantian Fang, Hong Fu

**Affiliations:** ^1^ Department of Gastric Surgery, Fudan University Shanghai Cancer Center, Shanghai, China; ^2^ Department of Oncology, Shanghai Medical College, Fudan University, Shanghai, China; ^3^ Department of Nursing, Fudan University Shanghai Cancer Center, Shanghai, China

**Keywords:** Situs inversus totalis, gastric cancer, surgical management, vascular anomalies, gastrectomy

## Abstract

**Background:**

Situs inversus totalis (SIT) is an uncommon disorder characterized by mirror-image anatomy, which can present unique challenges and potential vascular anomalies in surgical interventions, particularly in gastric cancer patients.

**Aims:**

We aim to delineate a rare case of gastric adenocarcinoma in a SIT patient and conduct a thorough review of the existing literature concerning surgical strategies, vascular anomalies, and outcomes observed across varied geographic locales and technological approaches.

**Methods:**

A thorough examination of a case involving a 39-year-old male SIT patient who underwent a successful distal gastrectomy with D2 lymph node dissection is presented alongside an expansive literature review. The review encompasses 47 articles, collating data on surgical approaches and vascular anomalies across 49 patients diagnosed with SIT and gastric cancer.

**Results:**

The patient underwent curative distal gastrectomy and Billroth II with Braun anastomosis within 95 minutes, incurring minimal intraoperative blood loss (100ml). Postoperative pathology confirmed moderately to poorly differentiated gastric adenocarcinoma (pT3N0M0), with no signs of recurrence or metastasis after 6 months of S-1 adjuvant chemotherapy. The literature review revealed vascular anomalies in approximately 20% of reported cases, accentuating its surgical significance. Noteworthy variations in surgical strategies, operative times, blood loss, and complications across different surgical modalities were observed, providing a comprehensive view into the practical management of such cases.

**Conclusion:**

Despite the inherent challenges associated with SIT, various surgical techniques can be successfully applied with meticulous preoperative planning and understanding vascular anomalies. This compilation of diverse surgical experiences across numerous documented cases seeks to provide a consolidated resource for refining surgical strategies and enhancing postoperative outcomes for gastric cancer patients with SIT, underscoring the imperativeness of further research in this niche domain.

## Introduction

Navigating through the complex world of Situs inversus totalis (SIT), we delve into the subtleties of this congenital condition and explore its relationship with gastric cancer management. SIT is characterized by a mirror-image transposition of the standard arrangement of visceral organs, sparking both academic intrigue and presenting tangible hurdles in surgical scenarios (1). This condition falls within the broader category of Situs Viscerum Inversus (SVI), which can be further divided into: “situs viscerum inversus partialis” (SVIP), a less common variant affecting only a single or a group of viscera, and SIT, our main focus, characterized by complete organ transposition and supported by pertinent literature (2). Despite its relatively rare occurrence, estimated at 1 in 10,000 individuals, SIT is often accidentally discovered due to its typically asymptomatic nature but becomes significantly relevant in surgical situations because of its unique anatomical arrangement (3, 4).

Globally, gastric cancer ranks as the fifth most prevalent cancer type and the third most deadly cause of cancer-related mortality (5). Despite advances in early detection, a significant proportion of gastric cancer patients are diagnosed at advanced stages, for which surgical resection remains the cornerstone of curative treatment. However, the management of gastric cancer in patients with SIT can be technically demanding due to the mirror-image anatomy, necessitating careful preoperative planning and adaptability in surgical techniques (6).

This study presents the case of a 39-year-old male patient with SIT who underwent successful distal gastrectomy with D2 lymph node dissection for gastric adenocarcinoma. In addition to the case report, the goal is to conduct an exhaustive review of the available literature, shedding light on the diverse surgical modalities utilized in managing gastric cancer in SIT patients. The surgical approaches range from Endoscopic Submucosal Dissection (ESD), a minimally invasive technique favored for early-stage cancers, to more traditional open surgical procedures (7). Modern surgical techniques, such as laparoscopic surgery and robotic surgery, are also taken into account given their growing prominence (8). Further extending our investigation’s breadth, we identified a variety of vascular anomalies within the scrutinized literature, which has the potential to offer an invaluable repository of data to comprehend the potential vascular variations in such uncommon cases better. The understanding of these anomalies holds pivotal clinical relevance in both preoperative planning and the intraoperative surgical procedure, steering surgeons clear of inadvertent trauma.

Through an amalgamation of practical clinical experience, as embodied in the case report, and theoretical understanding, as derived from the literature review, this study endeavors to augment the collective knowledge pool. By bringing to light the unique considerations and adaptations required in such cases, this study aims to enhance clinical outcomes and broaden the horizons of surgical intervention.

## Case report

**Figure 1:**
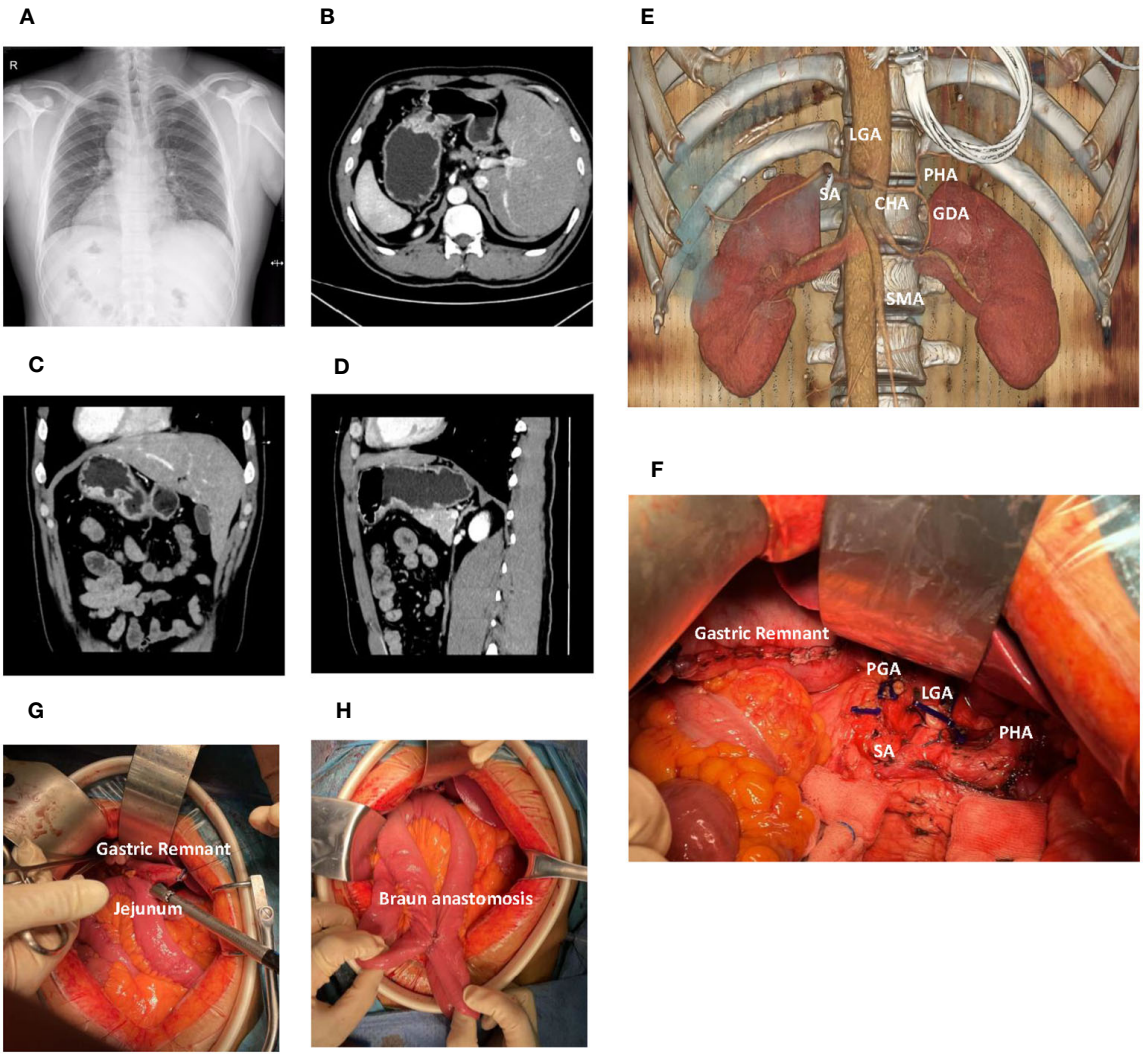
Comprehensive radiological and intraoperative visualization in Situs Inversus Totalis (SIT) accompanied by gastric cancer. **(A)** Chest X-ray demonstrates dextrocardia and mirror-image arrangement of the thoracic structures. **(B)** Axial computed tomography (CT) scan reveals a gastric lesion located in the body of the stomach. **(C)** Coronal CT image shows the gastric lesion in the body of the stomach. **(D)** Sagittal CT image further delineates the gastric lesion in the stomach body. **(E)** Three-dimensional vascular reconstruction depicts major vessels such as the celiac trunk and Superior Mesenteric Artery (SMA). **(F)** The surgical field following distal gastrectomy and D2 lymph node dissection, detailing the altered anatomy and resected regions. **(G)** The gastrojejunostomy utilizing a linear cutter stapler. **(H)** The Braun anastomosis. LGA, Left Gastric Artery; PGA, Post Gastric Artery; SA, Splenic Artery; CHA, Common Hepatic Artery; PHA, Proper Hepatic Artery; SMA, Superior Mesenteric Artery.

A 39-year-old male, who had no notable medical history or lifestyle-related risk factors, reported persistent upper abdominal discomfort for over three months. Chest X-ray revealed dextrocardia with mirror-image branching of the great vessels (**Figure 1A**). Gastroscopy performed at a local hospital identified an ulcer (8x12mm) on the posterior wall of the greater curvature of the stomach. Biopsy suggested a low-differentiated adenocarcinoma, and further Computed Tomography (CT) imaging confirmed gastric cancer with SIT (**Figures 1B-D**). To better understand the aberrant vascular distribution in the abdomen and aid in meticulous surgical planning, a three-dimensional reconstruction of the abdominal vessels was completed. No anomalies were detected apart from the mirroring inherent to SIT (**Figure 1E**).

The patient underwent distal gastrectomy with D2 lymph node dissection under general anesthesia, followed by Billroth II and Braun anastomoses (**Figures 1F-H**). Unique to this case, the SIT necessitated careful surgical planning and adaptation of standard procedures. The tumor, found in the gastric antrum (approximately 2x2cm in size), had serosal invasion and adhesion to the transverse mesocolon. No metastatic nodules were detected in the pelvic floor, abdominal wall, or mesentery. Careful and meticulous dissection was carried out, ensuring adherence to oncological principles despite the challenging SIT anatomy.

In this surgical procedure, a section of the transverse colon approximately 2cm from the tumor margin was resected. The omentum was freed along the transverse colon, extending from the splenic flexure to the hepatic flexure. The right gastroepiploic vessels at the base were ligated and lymph nodes beneath the pylorus were cleared. The omentum near the liver was severed, and the lymph nodes along the hepatoduodenal ligament (group 12) were cleared. The right gastric vessels at the base were ligated, and lymph nodes above the pylorus were cleared. The duodenum was cut 2cm below the pylorus, and the stump was sealed with a stapler. The seromuscular layer was sutured for reinforcement. The stomach was elevated and lymph nodes along the surface of the common hepatic artery (group 8) were cleared. The coronary vein was severed, and the left gastric artery at the root was ligated. Lymph nodes in groups 7, 9, and 11p were cleared. The left gastroepiploic vessels and a short gastric artery at the base were ligated, and lymph nodes from the greater curvature of the stomach to the intended site of stomach transection were cleared. Lymph nodes to the right of the cardia were cleared. The stomach was then transected using a linear stapler and the specimen was removed. The jejunum was elevated 30cm from the Treitz ligament, and a gastrojejunostomy was performed using a linear stapler. The anastomosis was located on the greater curvature of the stomach. The common opening of the stomach and jejunum was closed by continuous inverted suturing. A Braun anastomosis was additionally performed. The surgical field was rinsed and hemostasis confirmed. A drainage tube was placed along the upper edge of the pancreas and fixed after being drawn out from the right upper abdominal wall. After confirming that no instruments or gauze were left behind, the abdomen was closed layer by layer.

After the surgery, the duration of which was 95 minutes with an estimated blood loss of approximately 100ml, the pathological examination revealed a moderately to poorly differentiated adenocarcinoma of the gastric antrum, staged as pT3N0M0. He commenced a liquid diet on the third day postoperatively after the return of bowel function. The patient was discharged on the seventh day and was planned to prescribe S-1 oral chemotherapy as an adjuvant treatment for one year. Follow-up examinations were routinely carried out, and no notable recurrence or metastasis was identified at month 6 postoperatively.

## A review of surgical strategies for gastric cancer

**Figure 2:**
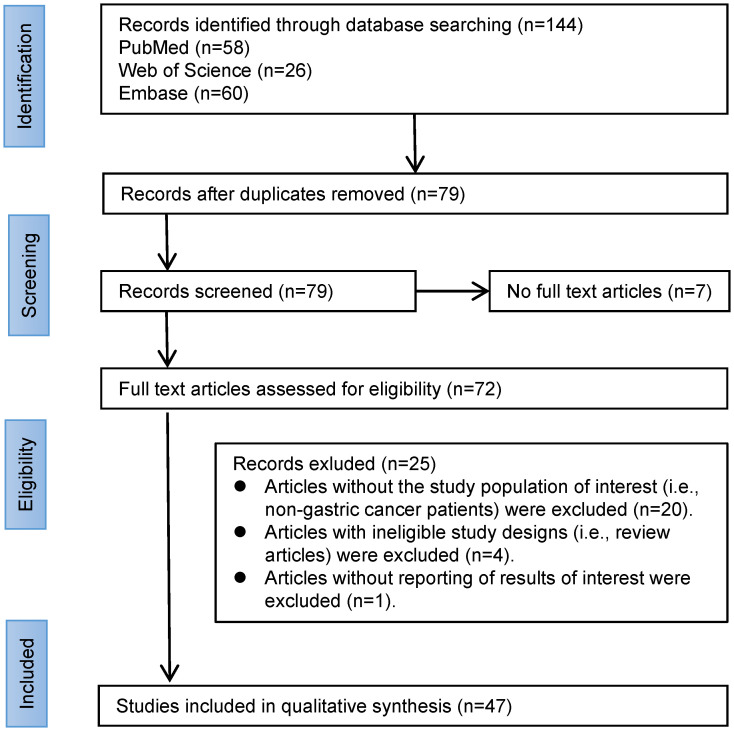
Literature review flowchart.

To glean information on surgical strategies for SIT in conjunction with gastric cancer, we performed a comprehensive search on PubMed, Web of Science (WOS), and Embase databases using the key terms ‘SIT’ and ‘gastric cancer’ (**Figure 2**). From the years 2000 to 2023, we retrieved a total of 132 records. After removing duplicates and literature without full text, we further screened the remaining 72 case reports. Only cases reporting surgical treatment of SIT combined with gastric cancer were included in this study. During the full-text screening phase, we excluded 25 articles, and finally, 47 articles were deemed eligible according to the inclusion criteria and were assessed.

**Figure 3:**
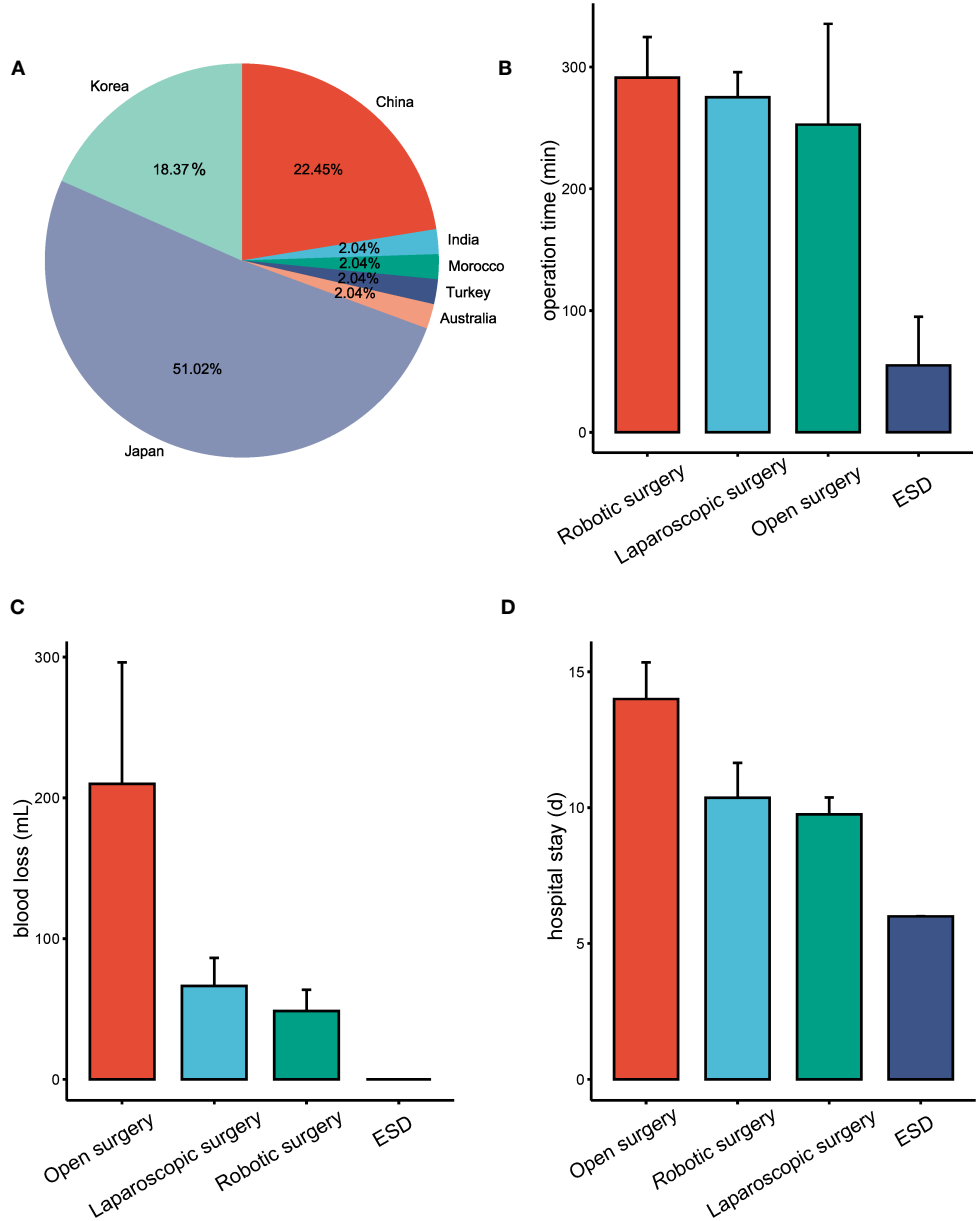
Overview of SIT coexistent with gastric cancer cases and treatment outcomes based on different surgical approaches. **(A)** Geographic distribution of reported cases of SIT combined with gastric cancer. **(B)** Surgical duration associated with different operative procedures in the treatment of patients with SIT coexistent with gastric cancer. **(C)** Estimated blood loss during various surgical procedures for patients with SIT combined with gastric cancer. **(D)** Length of hospital stay following different surgical procedures for SIT patients combined with gastric cancer.

The analysis of these 47 articles, along with our own study, accounts for a total of 49 patients diagnosed with SIT and gastric cancer (**Supplementary Data**). Among these, one case involved diffuse large B-cell lymphoma, with the remaining diagnosed with gastric adenocarcinoma. The median age was 63 years, ranging from 29 to 89 years, and included 35 males and 14 females. The geographical distribution showed one patient from Morocco, one from India, one from Turkey, one from Australia, eleven from China, nine from Korea, and twenty-five from Japan (**Figure 3A**).

Tumor locations varied, with 27 located in the lower (L) portion of the stomach, 10 in the middle (M) portion, and 12 in the upper (U) portion. All patients underwent surgical intervention, comprising 13 open abdomen procedures, 21 laparoscopic surgeries, 11 robotic-assisted or robotic surgeries, and 4 endoscopic surgeries, one of which proceeded to laparoscopic surgery post-endoscopic submucosal dissection (ESD).

The mean operation times for ESD, laparoscopic surgery, robotic surgery, and open abdomen procedures were 55min (15-95min), 275min (117-446min), 291min (180-451min), and 252min (95-375min), respectively (**Figure 3B**). Estimated blood loss was minimal in ESD, while laparoscopic surgery averaged 66ml (0-350mL), robotic surgery 43ml (0-150ml), and open abdomen procedures 210ml (100-380ml) (**Figure 3C**). Patients undergoing ESD stayed an average of 6.00 days, which was significantly shorter when compared to 9.84 days for laparoscopic surgery, 10.36 days for robotic surgery, and 14.00 days for open surgery (**Figure 3D**).

Complications were reported in a few patients: one experienced mechanical bowel obstruction following laparoscopic surgery (9), one presented with a pancreatic fistula and hepatic dysfunction post-robotic surgery (10), and one case involved poor postoperative wound healing (11).

**Figure 4:**
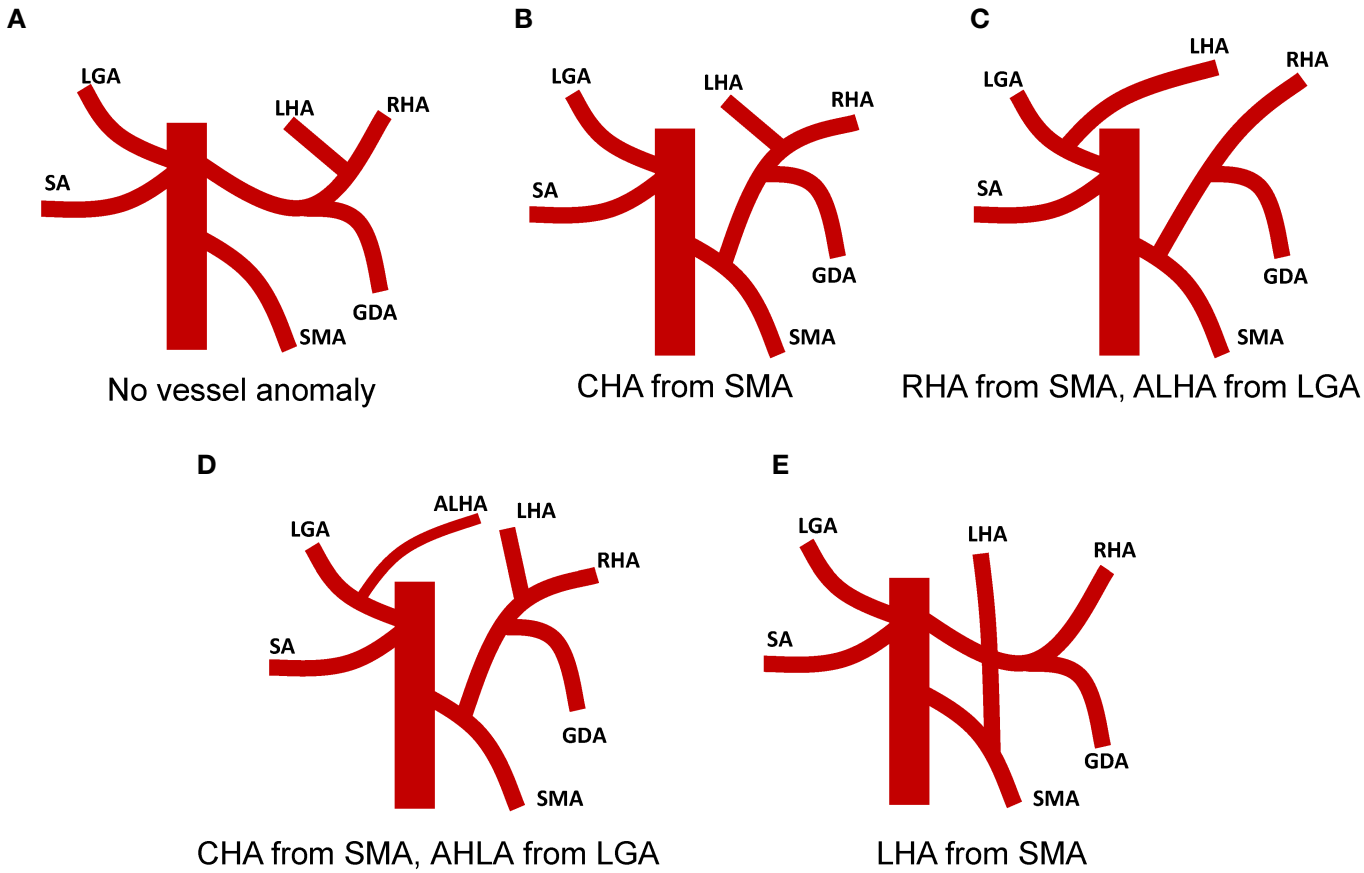
Vascular anomalies associated with surgical intervention in patients with Situs Inversus Totalis (SIT) coexistent with gastric cancer. **(A)** No vascular anomaly observed in the patient. **(B)** Patient with Common Hepatic Artery (CHA) originating from the Superior Mesenteric Artery (SMA). **(C)** Patient with Right Hepatic Artery (RHA) originating from the SMA and Accessory Left Hepatic Artery (ALHA) deriving from the Left Gastric Artery (LGA). **(D)** Patient with CHA originating from SMA and ALHA arising from LGA. **(E)** Patient with Left Hepatic Artery (LHA) branching from the SMA.

In our literature review of SIT concomitant with gastric cancer patients, we found noteworthy instances of vascular anomalies. Out of the 49 patients we reviewed, 10 reported vascular variations, offering valuable data to better comprehend the possible vascular anomalies in such rare cases (**Figure 4**). Among these 10 patients, four observed that the common hepatic artery (CHA) branched from the superior mesenteric artery (SMA)(**Figure 4B**) (12–15). Two patients had an unusual vascular presentation where the right hepatic artery (RHA) originated from the SMA, and the left hepatic lobe was supplied by the ALHA originating from the Left Gastric Artery (LGA)(**Figure 4C**) (4, 13). In one patient, the CHA arose from the SMA and an accessory left hepatic artery (ALHA) was noted to originate from the Left Gastric Artery (LGA)(**Figure 4D**) (16). Another patient presented a variant where the left hepatic artery branched from the superior mesenteric artery (**Figure 4E**) (17). This anomaly may require additional caution during surgical procedures to avoid unnecessary trauma. Moreover, one patient revealed a vascular anomaly where the right gastroepiploic artery (RGEA) was positioned above the right gastroepiploic vein (RGEV) (18). Finally, one patient presented with an azygous continuation of the interrupted inferior vena cava, direct hepatic vein drainage into the left atrium, and a preduodenal portal vein (19).

## Discussion

Managing gastric cancer in patients with SIT demands distinctive considerations due to the mirror-image anatomy of the abdominal organs. The present report outlines the case of a 39-year-old male with SIT diagnosed with gastric cancer and furnishes an extensive review of the literature that includes 47 articles documenting 49 similar cases. The presented case and the broader literature underscore the imperative for personalized surgical strategies for this exceptional patient group.

While the importance of thorough preoperative planning and understanding the unconventional, mirror-image anatomy inherent in SIT cannot be overstated, an equally pivotal aspect is the strategic decision-making involved in selecting a post-distal gastrectomy reconstruction method. Initially, Roux-en-Y (R-Y) reconstruction might appear advantageous due to its marginally lower risk of residual gastritis and bile reflux as compared to Billroth II (B-II), with no significant disparities in postoperative complications and mortality rates, as elucidated by existing research (20–22). Nonetheless, our predilection for B-II reconstruction, complemented with Braun anastomosis, despite the minutely reduced associated risks of R-Y, pivoted on three foundational considerations. Firstly, we targeted meticulous risk management by particularly circumventing the complexities and bleeding risks tied to jejunal vessel ligation in an SIT context (23). Secondly, we were committed to ensuring proficient gastric emptying, employing Braun anastomosis to offset potential delays sometimes associated with R-Y (21). Thirdly, in the pursuit of maintaining anatomical congruence, we opted for B-II with Braun anastomosis owing to its harmonious alignment with our center’s prevailing operative methodology. In our center, given the higher incidence of gastric atony and obstruction, experts have a pronounced preference for B-II with Braun anastomosis over R-Y reconstruction. The data from our center, which underscores this preference, will be elucidated in forthcoming research.

In our report, the patient with SIT and concurrent advanced-stage gastric cancer successfully underwent distal gastrectomy with D2 lymph node dissection. The integration of preoperative CT three-dimensional reconstruction and vascular modeling facilitated a profound understanding of anatomical intricacies, including lesion attributes, associations with adjacent tissues, and anomalous vascular patterns, thereby shaping the surgical strategy. This exemplifies that, despite the complexities inherent to SIT, meticulous preparation and surgical execution can manifest in desirable oncological outcomes (6).

Conducting surgeries in SIT patients with gastric cancer does indeed present unique challenges due to the mirror-image anatomy. Nonetheless, by understanding and adeptly applying the fascial anatomical layers and vascular sheath structures integral to gastric cancer surgery, these challenges can be effectively surmounted (24). Strategies like recognizing the landmark role of the pancreas, identifying fusion fascial spaces and utilizing microsurgical planes for vessel dissection can substantially enhance surgical success (25). The pancreas serves as a critical anatomical guide during radical gastrectomy, with numerous pertinent vessels coursing around it, including the left gastric artery, common hepatic artery, gastroduodenal artery, splenic artery, left gastric vein, and portal vein. Within the ambit of gastric cancer surgery, the vascular sheath - composed of connective tissues encapsulating and safeguarding the vessels - embodies a crucial anatomical layer (26). Surgeons dissect along this vascular sheath, enabling the identification of intersecting vessels, secure lymph node dissection, and reduction of bleeding from tissues such as adipose and lymph nodes. In the context of SIT, the surgical steps may need to be mirrored to accommodate the abnormal positions of the organs. For instance, steps that are typically performed on the right side may need to be performed on the left side, and vice versa. However, this does not change the basic steps or objectives of the surgery. While SIT may make the surgery more complex, successful gastrectomy for gastric cancer can still be achieved through proper surgical planning and techniques.

The major challenges include the difficulty in curative lymph node dissection, an increased propensity for intraoperative and postoperative bleeding, and the potential impact on organ function following vascular injury. Variations in the branching patterns of the CHA can present significant challenges in the dissection of lymph nodes around the SMA (12). This underscores the significance of preoperative imaging and planning to ensure curative resection while minimizing surgical morbidity. The potential for increased intraoperative and postoperative bleeding due to vascular anomalies cannot be underestimated. Unique vascular presentations, such as the RHA originating from the SMA and the left hepatic lobe being supplied by the ALHA from the LGA, necessitate superior surgical expertise to prevent inadvertent damage to these vessels (13, 16). The atypical positioning of the RGEA above the RGEV accentuates the necessity for meticulous dissection techniques, considering the unexpected vessel locations (18). Vascular injury could potentially lead to severe postoperative complications, particularly spleen ischemia and hepatic dysfunction. The numerous variants in the route of the left gastric vein, which may drain into the splenic vein, portal vein, or common hepatic vein, further complicate the surgical field. The left gastric vein carries a significant blood flow and if injured, could cause substantial hemorrhage, complicating the surgical procedure.

Historically, open abdominal surgery has been the go-to treatment for gastric cancer, owing to its wide surgical field that enables extensive lymphadenectomy. Yet, as demonstrated by our review, it typically involves larger incisions that can potentially lead to augmented blood loss and prolonged recovery periods. In contrast, laparoscopic surgery offers a less invasive alternative characterized by smaller incisions, diminished blood loss, and potentially shorter hospital stays (27). However, the mirror-image anatomy of SIT patients demands advanced surgical expertise and could restrict the utility of this method, particularly in advanced cases requiring extensive lymphadenectomy or when dealing with larger tumors. Our literature review also hinted that robotic-assisted surgery might serve as a promising alternative for managing gastric cancer in SIT patients (28). Owing to its 3D magnified view and enhanced ergonomics, the challenges posed by the mirror-image anatomy of SIT can be significantly reduced. Yet, a cautious approach is warranted due to potential complications, as highlighted by a case in our review reporting postoperative pancreatic fistula and hepatic dysfunction (10). For gastric cancer that is limited to the mucosa or submucosa and carries a low risk of lymph node metastasis, endoscopic surgery, particularly ESD, might be a suitable option. ESD provides a minimally invasive procedure with minimal blood loss and shorter operation duration (29). However, its applicability may be limited in advanced stages of gastric cancer.

Surgery for gastric cancer in patients with SIT presents unique challenges due to the mirror-image anatomy. However, strategies such as recognizing key anatomical landmarks, identifying fusion fascial spaces, and utilizing microsurgical planes for vessel dissection can significantly improve surgical success. The choice of surgical approach ought to be personalized, taking into account the specifics of the disease, the patient’s health status, and the surgeon’s expertise. By adopting these strategies, surgeons can effectively manage gastric cancer in SIT patients, leading to improved patient outcomes.

## Data availability statement

The original contributions presented in the study are included in the article/**Supplementary Material**. Further inquiries can be directed to the corresponding author.

## Ethics statement

The research was approved by the Ethics Committee of Fudan University Shanghai Cancer Center. Written informed consent was obtained from the individual(s) for the publication of any potentially identifiable images or data included in this article.

## Author contributions

HF designed the study, conducted the literature review, and drafted the manuscript. BS performed the surgical procedure on the patient and provided critical insights for the case study. PX oversaw the study design and approved the final version of the manuscript. PK assisted in surgical procedures, conducted post-operative follow ups and contributed to the manuscript's methodology section. YF assisted in data interpretation, ensured the accuracy of clinical details and contributed to discussions and manuscript finalization. All authors read and approved the final manuscript.
