# Supplementary material for: Gastric adenocarcinoma in Situs inversus totalis: a case study and literature review
Source: Front Oncol. 2023 Oct 25;13:1238467. doi: 10.3389/fonc.2023.1238467 (PMC10635521; doi:10.3389/fonc.2023.1238467)
Supplement: Supplementary file 1 [file Table_1.docx]

| **Table S1** The features of laparoscopic gastrectomy for situs inversus totalis patients with gastric cancer. | | | | | | | | | | | | | |
| --- | --- | --- | --- | --- | --- | --- | --- | --- | --- | --- | --- | --- | --- |
| **Author** | **Year** | **Regions** | **Age** | **Sex** | **Tumour location** | **Vessel anomaly** | **Surgical strategy** | **Lymph node dissection** | **TNM**  **stage** | **Operative time (min)** | **Blood loss (mL)** | **Hospital stay (D)** | **Post surgical complications** |
| Iwamura et al. | 2001 | Japan | 71 | Female | U | azygous continuation of the interrupted inferior vena cava, direct drainage of hepatic vein to left atrium,preduodenal portal vein | TG+lowanterior resection of rectum | ND | 1a | ND | ND | ND | no |
| Murakami et al. | 2003 | Japan | 51 | Female | U |  | TG+cholecystectomy | D2 | ND | ND | ND | ND | no |
| Haruki et al. | 2010 | Japan | 81 | Female | L |  | bypass | no | 4 | ND | ND | ND | poor postoperative wound healing |
| Fujikawa et al. | 2013 | Japan | 60 | Female | M |  | LADG | D1+ | 1a | 234 | 5 | 8 (after second operation) | mechanical obstruction |
| Ye et al. | 2015 | China | 60 | Female | L |  | LADG | D2 | 2b | 230 | 50 | 8 | no |
| Isobe et al. | 2015 | Japan | 79 | Female | M |  | TG+cholecystectomy | D2 | 3c | 288 | 150 | 19 | no |
| Zhu et al. | 2015 | China | 66 | Female | L |  | DG | D2 | 3a | ND | ND | ND | no |
| Alhossaini et al. | 2017 | Korea | 52 | Female | L |  | RADG | D2 | 1a | 195 | 30 | 5 | no |
| Aisu et al. | 2018 | Japan | 64 | Female | M |  | RADG | D1+ | 1a | 451 | 150 | 18 | Hepatopathy & pancreatic fistula |
| Namikawa et al. | 2018 | Japan | 66 | Female | M |  | TG | D2 | 1 | 375 | 380 | 14 | no |
| Harada et al. | 2021 | Japan | 63 | Female | U |  | LTG | D2 | 1b | 422 | 30 | 14 | no |
| Takeno et al. | 2021 | Japan | 71 | Female | U |  | RAPG | D1+ | 1a | 448 | 45 | 10 | no |
| Lamture et al. | 2022 | India | 48 | Female | L |  | DG+transverse colon | ND | ND | ND | ND | 14 | no |
| Lee et al. | 2023 | Korea | 79 | Female | M |  | LTG | D2 | 2b | 269 | ND | 14 | no |
| Park et al. | 2005 | Korea | 58 | Male | L |  | DG | D1+ | 1a | ND | ND | 15 | no |
| Tsutsumi et al. | 2007 | Japan | 73 | Male | L | CHA from SMA | DG | D1+ | 1a | ND | ND | ND | no |
| Benjelloun et al. | 2008 | Morocco | 70 | Male | L |  | DG | D2 | 3a | ND | ND | ND | no |
| Futawatari et al. | 2010 | Korea | 53 | Male | L |  | LADG | D1+ | 1a | 300 | 350 | 12 | no |
| Seo et al. | 2011 | Korea | 60 | Male | L |  | LADG+cholecystectomy | D1+ | 1a | 200 | 70 | 7 | no |
| Kim et al. | 2012 | Korea | 47 | Male | M |  | RADG | D1+ | 3b | 300 | ND | 8 | no |
| Pan et al. | 2012 | China | 52 | Male | U |  | proximal | D2 | ND | ND | ND | 15 | no |
| Min et al. | 2013 | Korea | 68 | Male | L |  | LDG | D1+ | 1a | 117 | 50 | 5 | no |
| Min et al. | 2013 | Korea | 52 | Male | L | CHA from SMA | LADG | D1+ | 1b | 220 | 100 | 8 | no |
| Sumi et al. | 2014 | Japan | 42 | Male | L | LHA from SMA | ESD+LDG | D1+ | 1b | 313 | 90 | 10 | no |
| Morimoto et al. | 2015 | Japan | 58 | Male | M |  | LATG | D1+ | 1a | 359 | 90 | 7 | no |
| Kigasawa et al. | 2017 | Japan | 40 | Male | L |  | LADG | D1+ | 1a | 284 | 40 | ND | no |
| Suh et al. | 2017 | Korea | 50 | Male | L |  | LDG | D2 | 1 | 180 | ND | 10 | no |
| Cao et al. | 2017 | China | 60 | Male | U | RHA from SMA, ALHA from LGA | RATG | D2 | 2b | ND | ND | 8 | no |
| Dai et al. | 2018 | China | 53 | Male | L |  | DG | D2 | 3a | 180 | 50 | 5 | no |
| Gündeş et al. | 2018 | Turkey | 50 | Male | L |  | DG | D1 | 1a | 150 | 100 | 8 | no |
| Shibata et al. | 2018 | Japan | 79 | Male | U | RGEA above RGEV | LTG | D2 | 2b | 232 | 110 | 10 | no |
| Miyaoka et al. | 2018 | Japan | 72 | Male | L |  | ESD | no | 1a | ND | ND | ND | no |
| Ojima et al. | 2019 | Japan | 80 | Male | L |  | RADG | D2 | 1 | 260 | 20 | 14 | no |
| Xue et al. | 2019 | China | 61 | Male | L |  | DG | D2 | 4 | ND | ND | 14 | no |
| Koyama et al. | 2020 | Japan | 74 | Male | L |  | ESD | no | 1a | 15 | ND | ND | no |
| Horikawa et al. | 2020 | Japan | 83 | Male | L |  | ESD | no | 1a | ND | ND | 6 | mucosal laceration & bleeding |
| Abbey et al. | 2021 | China | 69 | Male | L |  | RADG | D2 | 3b | 205 | 20 | 15 | no |
| Namikawa et al. | 2021 | Japan | 74 | Male | M | CHA from SMA | LDG | D2 | 1 | 335 | 20 | 12 | no |
| Yoshimoto et al. | 2021 | Japan | 84 | Male | U |  | RATG | D2 | 3a | ND | 30 | 13 | no |
| Sivakumar et al. | 2021 | Australia | 29 | Male | U |  | LTG | ND | 1a | 392 | ND | 6 | no |
| Jian et al. | 2021 | China | 53 | Male | U |  | LRPG | D2 | 1 | ND | ND | 14 | no |
| Fukuda et al. | 2022 | Japan | 89 | Male | U |  | ESD | no | 1a | 95 | 0 | 6 | no |
| Katano et al. | 2022 | Japan | 62 | Male | U | CHA from SMA, ALHA from LGA | RAPG | D2 | 3b | 296 | 0 | 11 | no |
| Fujita et al. | 2022 | Japan | 67 | Male | L | CHA from SMA | LDG | D2 | 3a | 446 | 3 | 14 | no |
| Sato et al. | 2022 | Japan | 72 | Male | M |  | LDG | D2 | 3b | 323 | 10 | 10 | no |
| Sagawa et al. | 2022 | Japan | 64 | Male | L |  | RADG | D1+ | 1a | 286 | 44 | 7 | no |
| Zhu et al. | 2023 | China | 63 | Male | L | RHA from SMA, ALHA from LGA | LADG | D2 | 1b | ND | 0 | 10 | no |
| Huang et al. | 2023 | China | 58 | Male | L |  | LADG | D2 | 3 | 220 | 10 | 8 | no |
| Present case | 2023 | China | 39 | Male | M |  | DG | D2 | 2a | 95 | 100 | 7 | no |
| U: Upper-third of the stomach, M: Middle-third of the stomach, L: Lower-third of the stomach, CHA: Common Hepatic Artery, SMA: Superior Mesenteric Artery，LHA: Left Hepatic Artery，RHA: Right Hepatic Artery, ALHA: Accessory Left Hepatic Artery, RGEA:Right Gastroepiploic Artery, RGEV: Right Gastroepiploic Vein, DG: Distal Gastrectomy, TG: Total Gastrectomy, LADG: Laparoscopy-Assisted Distal Gastrectomy, LATG: Laparoscopy-Assisted Total Gastrectomy, LDG: Laparoscopy-assisted Distal Gastrectomy, LTG: Laparoscopy-assisted Total Gastrectomy, RADG: Robot-Assisted Distal Gastrectomy, RAPG: Robot-Assisted Proximal Gastrectomy, RATG: Robot-Assisted Total Gastrectomy, ESD: Endoscopic Submucosal Dissection, ND: Not described. | | | | | | | | | | | | | |
